# Gender Differences in Patients with Advanced Heart Failure: A Secondary Data Analysis of the ENABLE CHF-PC Randomized Clinical Trial

**DOI:** 10.1177/26892820251396380

**Published:** 2025-11-17

**Authors:** Lindsay Aaron-Wade, Rachel Wells, Andres Azuero, Shena Gazaway, J. Nicholas Odom, Marie Bakitas, Deborah Ejem

**Affiliations:** School of Nursing, University of Alabama at Birmingham, Birmingham, Alabama, USA.

**Keywords:** advanced heart failure, gender differences, palliative care, quality of life

## Abstract

**Background:**

Women appear to be underrepresented in heart failure and palliative care research. Given this underrepresentation, their unique characteristics, needs, and outcomes require further investigation.

**Methods:**

A secondary analysis of baseline data of the Educate, Nurture, Advise, Before Life Ends Comprehensive Heartcare for Patients and Caregivers study, a randomized clinical trial of Deep South patients ≥50 years of age with advanced HF. Differences in sociodemographics and measures of quality of life and mood between female and male patients were examined using bivariate tests and effect-size measures.

**Results:**

Statistically significant gender differences were observed with females reporting poorer quality of life-The Kansas City Cardiomyopathy Measure (49.55 ± 20.04 vs. 55.37 ± 21.52, *d* = 0.28, p-adj = 0.01), Patient-Reported Outcome Measurement Information System (PROMIS) Global Mental Health (44 ± 8.02 vs. 46.53 ± 9.01, *d* = 0.30, p-adj = 0.007), and PROMIS Global Physical Health (37.37 ± 7.65 vs. 39.2 ± 8.20, *d* = 0.23, p-adj = 0.034) and Hospital Anxiety and Depression Scale anxiety (6.9 ± 4.38 vs. 4.72 ± 3.89, *d* = 0.53, p-adj = 0.003) compared with men at baseline.

**Conclusion:**

Further investigation of gender differences is necessary to improve outcomes and inform the refinement of PC-HF interventions for females.

## Introduction

As of 2023, the heart failure (HF) prevalence of adults in the United States is about 1.9% to 2.8%; this number is higher among older patients and is expected to increase by 8.5% for those age 65–70.^1^ HF patients often experience debilitating symptoms such as dyspnea, peripheral edema, and orthopnea, which can limit their ability to perform activities of daily living.^2^ Palliative care (PC)^3^ for HF patients is intended to alleviate symptoms and improve quality of life (QOL).^4–7^ Despite recommendations, HF remains complex to manage due to unpredictable symptoms and lack of definitive diagnostic indicators.^8^ This complexity is often worse in advanced HF, as individuals may not qualify for advanced therapies and must rely on guideline-directed medical therapy.^4–10^

While HF affects both genders, inequities persist across the care continuum (e.g., clinical trial representation, lower rate of HF medication use).^11^ However, for PC to deliver universal benefits, gender inequities that influence treatment should be identified.^4^ Women have been historically underrepresented in research due to trial design, system barriers, and reproductive age concerns, resulting in treatment guidelines that utilize male-centered data.^11–13^ This is especially problematic as the incidence of HF with a preserved ejection fraction (HFpEF), a common subtype among women, increases.^14–16^ Women with HF are more likely to face challenges such as caregiver burden and symptom dismissal, which can contribute to poorer physical and emotional health.^10,17^

To explore these differences further, a secondary data analysis was conducted examining gender differences in QOL, mood, and mental health among adults with advanced HF who participated in the Educate, Nurture, Advise, Before Life Ends Comprehensive Heartcare for Patients and Caregivers (ENABLE CHF-PC) study.

## Theory

This study is guided by Wilson and Cleary’s Model of Quality of Life,^18^ which links clinical and health-related variables to overall QOL through a series of causal pathways, including biological and physiological variables, symptom status, functional status, and general health perceptions.^18^ The model also incorporates individual and environmental characteristics, which can influence the domain relationships.

## Materials and Methods

This is a secondary data analysis of baseline data from the ENABLE CHF-PC RCT, which tested the efficacy of a nurse-led, telehealth PC intervention.^19^ For this secondary analysis, we assessed differences between female and male baseline data in selected sociodemographics, QOL (Kansas City Cardiomyopathy Questionnaire and Functional Assessment of Chronic Illness Therapy–Palliative-14), mood (Hospital Anxiety and Depression Scale), and health-related QOL (PROMIS-Global Health).^20–23,24^ The study was approved by the University of Alabama at Birmingham and the Birmingham Veterans Affairs Medical Center Institutional Review Boards.

## Sample and Setting

Participants were recruited from two tertiary medical centers in the Deep South. Eligible patients met the following inclusion criteria: (1) English-speaking; (2) ≥ 50 years old; and clinician-documented New York Heart Association class III or IV, or American College of Cardiology/American Heart Association stage C or D HF. Patients who met eligibility and expressed interest provided written informed consent and completed a baseline assessment via telephone.

## Measures

### The Kansas city cardiomyopathy measure

The Kansas City Cardiomyopathy Measure (KCCQ) is a validated, self-administered 23-item questionnaire assessing QOL in patients with HF, with Cronbach’s alpha values ranging from 0.62 to 0.95, and six subscales demonstrating acceptable internal consistency (alpha values ≥ 0.78).^20^ The subscales include physical limitation, symptom burden and frequency, social limitation, QOL, and an overall summary.

### Hospital anxiety and depression scale

The HADS is a 14-item self-report measure developed to identify anxiety and depression and has demonstrated good reliability, with Cronbach’s alpha values ≥0.67 across various studies.^21^ The anxiety and depression subscales each have seven items.

### Patient-reported outcome measurement information system (PROMIS) global health

PROMIS Global Health is a 10-item patient-reported measure assessing physical and mental health-related QOL.^22,23^ It has shown adequate internal consistency, with Cronbach’s alphas of ≥0.79.^22,23^

### Data analysis

Comparisons between gender groups were conducted using descriptive statistics, T-tests, Chi-square tests, and measures of effect size (Cohen’s *d* and Cramer’s *V*), which were interpreted using Cohen’s guidelines.^25^ A false discovery rate adjustment^26^ was applied to account for multiple exploratory inferences and was set at 10% using the Benjamini-Hochberg procedure. This approach first ranks all individual *p*-values from smallest to largest, then calculates an adjusted *p*-value for each by considering its rank among all tests. It is designed to control the rate of false positive detections. Exploratory comparisons between gender groups were conducted for selected item-level scores of patient-reported outcomes using Cohen’s *d*.

## Results

Among the 415 participants, the mean age was 63.8 ± 8.6 years. Most identified as Black/African American (*n* = 226; 54.5%), urban dwelling (*n* = 307; 74%), and married or partnered (*n* = 201; 48.4%). Over half of the participants (52.8%) had a reduced ejection fraction (EF) (<49). Among female participants (*n* = 194), the mean age was 63.9 ± 8.8 years; Black/African American (*n* = 107; 55.2%); and urban dwelling (*n* = 151; 77.8%). One-third were married or partnered (*n* = 66; 34%), and 62.2% had HFpEF (EF >50).

As shown in Table 1, compared with males, females were significantly less likely to be partnered (34% vs. 61.1%, *v* = 0.29, p-adj = 0.003) and less likely to have a reduced EF (66.1% vs. 37.8%, *v* = 0.282, p-adj = 0.003). At baseline, females reported significantly poorer QOL as measured by the KCCQ compared with males (49.6 ± 20.0 vs. 55.4 ± 21.5, Cohen’s *d* = 0.28, p-adj = 0.01). Similarly, females reported lower PROMIS Global Mental Health (44 ± 8.0 vs. 46.53 ± 9.0, *d* = 0.30, p-adj = 0.007) and PROMIS Global Physical Health (37.4 ± 7.7 vs. 39.2 ± 8.2, *d* = 0.23, p-adj = 0.034). In addition, females reported significantly greater anxiety symptoms on the HADS compared with males (6.9 ± 4.38 vs. 4.72 ± 3.89, *d* = 0.53, p-adj = 0.003). There were no statistically significant between-group differences in HADS Depression scores (7.0 ± 3.6 vs. 6.5 ± 3.6, *d* = 0.14, p-adj = 0.227).

**Table 1.** Demographics and Between Group Differences

Characteristic	All (*n* = 415)	Female (*n* = 194)	Male (*n* = 221)	Effect size^b^	Test of difference		Effect size interpretation
	*n* (%)	*n* (%)	*n* (%)	*V* or *d* (95% CI)	*P*	Adjusted *P*^d^	
Age, mean (SD)	63.8 (8.6)	63.9 (8.8)	63.7 (8.4)	*d* = 0.02 (−0.17, 0.21)	0.858	0.934	Trivial
Race				0.08 (0, 0.14)	0.293	0.352	Small
Black/African American	226 (54.5%)	107 (55.2%)	119 (53.8%)				
White	184 (44.3%)	83 (42.8%)	101 (45.7%)				
Other	5 (1.2%)	4 (2.1%)	1 (0.5%)				
Urban residency	307 (74%)	151 (77.8%)	156 (70.6%)	0.08 (0, 0.17)	0.093	0.14	Small
Marital status				0.29 (0.2, 0.37)	<0.001	0.003	Medium
Never married	51 (12.3%)	33 (17%)	18 (8.2%)				
Married/partnered	201 (48.4%)	66 (34%)	135 (61.1%)				
Divorced/separated	108 (26.1%)	58 (29.9%)	50 (22.7%)				
Widowed	54 (13%)	37 (19.1%)	17 (7.7%)				
NYHA Status Class III	401(96.6%)	188 (96.9)	213 (96.4%)	0.02 (0, 0.09)	0.934	0.934	Trivial
Ejection fraction (EF)^a^, mean (SD)	41.1 (15.82)	45.8 (14.8)	37 (15.6)^c^	*d* = 0.58 (0.38, 0.77)	<0.001	0.003	Medium
EF <50%	219 (52.9%)	73 (37.8%)	146 (66.1%)	0.282 (0.19, 0.36)	<0.001	0.003	Medium
	Mean (SD)	Mean (SD)	Mean (SD)^c^	D			
QOL (KCCQ overall summary)	52.64(21.01)	49.55 (20.04)	55.37 (21.52)	0.28 (0.08, 0.47)	0.005	0.01	Small to medium
Depression (HADS anxiety)	6.71(3.61)	6.97 (3.57)	6.48 (3.64)	0.14 (−0.06, 0.33)	0.170	0.227	Small
Anxiety (HADS depression)	5.75(4.26)	6.9 (4.38)	4.72 (3.89)	0.53 (0.34, 0.72)	<0.001	0.003	Medium
Global physical health (PROMIS)	38.35(7.99)	37.37 (7.65)	39.2 (8.20)	0.23 (0.04, 0.42)	0.020	0.034	Small
Global mental health (PROMIS)	45.34(8.65)	44 (8.02)	46.53 (9.01)	0.30 (0.1, 0.48)	0.003	0.007	Small to medium

^a^ Ejection fraction—measurement of blood pumped from left ventricle.

^b^ Cramer’s *V* and Cohen’s *d* for categorical and numerical characteristics, respectively.

^c^ Missing data on one participant.

^d^ False Discovery Rate adjustment.

Magnitude of effect sizes (Cohen, 1988).

Cohen’s *d*: small ∼0.2, medium ∼0.5, large ∼0.8.

Cramer’s *V* (comparing 2 groups): small ∼0.1, medium ∼0.3, large ∼0.5.

HADS, Hospital Anxiety and Depression Scale; KCCQ, The Kansas City Cardiomyopathy Measure; PROMIS, Patient-Reported Outcome Measurement Information System; QOL, quality of life.

While some group differences were statistically significant, many of the effect sizes were small, suggesting limited clinical impact. In women, anxiety symptoms did present a medium effect size, which could prove meaningful in clinical practice.

The exploratory examination of differences in individual item scores between females and males in the items composing PROMIS Global Physical Health, PROMIS Global Mental Health, HADS Anxiety, and KCCQ Overall Summary (Supplementary Table S1) indicated that the *most considerable* differences were related to anxious thoughts and mobility limitations, with men reporting, on average, lower levels of both than women.

## Discussion

Guided by Wilson and Cleary’s theory of Quality of Life, the purpose of this secondary analysis was to examine gender differences in QOL, mood, and mental health and illuminate key differences to guide future gender-sensitive PC interventions.

Although most baseline characteristics were similar across gender groups, notable differences emerged that may help explain disparities in health outcomes, as women were more likely to be unpartnered and to have HFpEF. While the women were more likely to be unpartnered, this does not imply reduced social support; it raises the question of whether social support should be further investigated in the population. Limited clinical management options in the setting of HFpEF may contribute to lower QOL and higher depressive symptoms observed among women; in addition, the women may have been more advanced in their disease. According to Wilson and Cleary’s model, both social support (environmental factor) and symptom burden (biological/symptoms status) directly influence QOL. Our findings support this relationship and suggest that gender-specific variations in these domains may partially explain observed differences in lived experience (i.e., QOL, mental health, and depressive symptomology).^27^ Societal gender roles may influence the care and support females receive, which aligns with current trends in the literature regarding gender bias, and PC is recommended to address these challenges and support patients.^27^

Women in this study reported worse outcomes across patient-reported measures, including physical and mental health, global well-being, and depressive symptoms. Previous studies on women with HF have noted that those diagnosed with HFpEF experience a poorer QOL than men, reporting lower KCCQ scores and a worse functional status.^28,29^ While depression symptoms did not differ significantly, the magnitude of the difference in anxiety scores (Cohen’s *d* = 0.53) was notable; clinical significance should be determined in future studies. Most effect sizes were noted to be small to moderate. Even minimal findings raise important questions about how societal expectations, caregiving roles, and under-recognition of women’s symptoms may shape their health trajectories, especially for patients with limited treatment options. While the findings should be interpreted cautiously, they prove to be important for future hypothesis generation. The persistence of gender bias in HF diagnosis and treatment—particularly for HFpEF—may further contribute to these disparities, especially if these women lack social support.^11,12^ One systematic review pointed out that women with heart disease face challenges such as symptom dismissal and caregiver burden, which could affect their overall health.^30^

These results point to the role of early PC in addressing gender-specific needs in HF. PC offers a framework to help patients manage complex symptoms, navigate care decisions, and improve QOL, particularly in conditions like HFpEF, where curative options are limited. Interventions tailored to women’s unique challenges, such as social isolation, caregiver strain, and mental health concerns, could help close gender gaps. Our findings highlight a broader need to integrate psychosocial and gender-sensitive assessments in routine HF care, particularly in under-resourced and diverse settings.

## Limitations

This study has a few limitations. As a secondary data analysis, the study is limited to variables collected in the parent trial and cannot assess factors such as participants’ understanding of their HF diagnosis and prognosis, which may influence QOL and mental health. While the dataset was racially diverse and included a large sample of older adults with advanced HF, it was not designed to examine gender differences, gender identity, or social factors specifically. Future analyses could examine whether QOL differences exist between HFpEF and HFrEF and whether gender is a modifier. As such, the findings should be interpreted as exploratory and hypothesis-generating. Although the parent study was adequately powered, stratified analyses by gender may be underpowered for detecting smaller subgroup differences. Finally, while this study focused only on baseline data to understand the population before the intervention, it would be beneficial to examine the same data over time to determine whether observed gender differences persisted or evolved differently. Future studies on gender differences would benefit from examining QOL and mental health in the setting of HF over the long term to identify points along the trajectory where interventions could be more effective.

## Conclusion

HFpEF is a growing public health challenge, especially among older women, who remain underrepresented in HF clinical trials. While this study adds to an increasing body of evidence that women with advanced HF, particularly those with HFpEF, may experience poorer QOL, more depressive symptoms, and worse mental and physical health than men, more research is necessary to understand the potential gender differences in HF fully. Addressing gender disparities in symptom burden, treatment access, and social support could prove critical to improving outcomes.^31,32^ While the study cannot establish that gender-sensitive PC improves QOL, the findings do highlight the need to design and test interventions, with future studies to determine causality.

## Implications

These findings suggest several key directions for improving care of older women with advanced HF, particularly with HFpEF. While other studies have shown that QOL can be improved with PC interventions, women did not always show clinically significant effects; however, differences could become clinically significant if qualitative components are added.^33^ Interventions should prioritize social support strategies for unpartnered women who may lack informal caregivers. Implementation of periodic depression and anxiety screening and psychosocial assessment could prove beneficial. Models such as nurse or lay-navigator programs and structured peer support groups may help fill critical gaps. Given limited therapeutic options for HFpEF, PC should be introduced early to manage symptom burden and address mental health needs. Utilizing a multidisciplinary team approach, including mental health specialists, could better support the women. Health systems should explore how home-based services—such as visiting nurse programs or community health worker outreach—can be deployed to support women aging with HF. Finally, future research must continue to examine gender-specific pathways in HF to inform targeted, equitable interventions.
