# Supplementary material for: Gender Differences in Patients with Advanced Heart Failure: A Secondary Data Analysis of the ENABLE CHF-PC Randomized Clinical Trial
Source: Palliat Med Rep. 2025 Nov 17;6(1):595–600. doi: 10.1177/26892820251396380 (PMC12670693; doi:10.1177/26892820251396380)
Supplement: Supplementary Data [file 26892820251396380_supplementary_data.docx]

**Authorship Confirmation**

LAW: Conceptualization, Writing- Original Draft Preparation; RW: Conceptualization, Resources, Supervision Data Curation, Writing- Reviewing and Editing; SG: Conceptualization, Supervision, Writing- Reviewing and Editing; AA: Formal Analysis, Supervision, Writing- Reviewing and Editing; Validation; JNO: Investigation, Supervision, Writing- Reviewing and Editing; MB: Investigation, Funding Acquisition, Resources, Supervision, Writing- Reviewing and Editing; DE: Investigation, Resources, Supervision, Writing- Reviewing and Editing.

**Authors’ disclosure**

The authors disclose no real or perceived vested interests in this article that might be considered a conflict of interest.

**Funding**

The authors disclose that no funding has been received in relation to this manuscript.
