# Supplementary material for: Gender Differences in Patients with Advanced Heart Failure: A Secondary Data Analysis of the ENABLE CHF-PC Randomized Clinical Trial
Source: Palliat Med Rep. 2025 Nov 17;6(1):595–600. doi: 10.1177/26892820251396380 (PMC12670693; doi:10.1177/26892820251396380)
Supplement: Supplementary Table S1 [file 26892820251396380_supplementary_table_s1.docx]

**Supplemental Table**. Items with a standardized mean score difference of at least 0.2 in absolute value (a small effect size) between females and males in the PROMIS Global Physical Health, PROMIS Global Mental Health, HADS Anxiety, and KCCQ Overall Summary scales. Scores for 35 items were examined but only results for 20 items are shown (those with |d|≥0.2).

| **Instrument and Item** | | **Females (n=194)** | | **Males (n=221)** | | **Difference** |
| --- | --- | --- | --- | --- | --- | --- |
|  |  |  |  |  |  | **Fem. - Male** |
|  |  | **Mean** | **SD** | **Mean** | **SD** | **Cohen's d** |
| **PROMIS Global Physical Health (4 items total, item score range = 1-5, higher scores = higher perceived health-related QOL)** | |  |  |  |  |  |
|  | To what extent are you able to carry out your everyday physical activities such as walking, climbing stairs, carrying groceries, or moving a chair? | 2.61 | 1.1 | 2.84 | 1.16 | 0.2 |
|  | In the past 7 days, how would you rate your fatigue on average? (reverse coded) | 3.03 | 0.91 | 3.24 | 1 | 0.22 |
| **PROMIS Global Mental Health (4 items total, item score range = 1-5, higher scores = higher perceived health-related QOL)** | |  |  |  |  |  |
|  | In general, how would you rate your mental health, including your mood and your ability to think? | 3.18 | 1.09 | 3.4 | 1.13 | 0.2 |
|  | In the past 7 days, how often have you been bothered by emotional problems such as feeling anxious, depressed or irritable? (reverse coded) | 3.53 | 1.17 | 3.96 | 1.18 | 0.37 |
| **HADS Anxiety (7 items total, item score range = 0-3, higher scores = higher distress)** | |  |  |  |  |  |
|  | I feel tense or "wound up." | 1.18 | 1.01 | 0.83 | 0.95 | -0.35 |
|  | I get a sort of frightened feeling as if something awful is about to happen. | 1.01 | 0.96 | 0.58 | 0.82 | -0.48 |
|  | Worrying thoughts go through my mind. | 1.21 | 1.01 | 0.78 | 0.92 | -0.45 |
|  | I can sit at ease and feel relaxed (reverse coded) | 0.98 | 0.85 | 0.76 | 0.83 | -0.26 |
|  | I get a sort of frightened feeling like butterflies in the stomach. | 0.82 | 0.88 | 0.54 | 0.75 | -0.34 |
|  | I get sudden feelings of panic. | 1.01 | 0.96 | 0.7 | 0.92 | -0.33 |
| **KCCQ Overall Summary (20 items total, item score range = 1-5, higher scores = better health status)** | |  |  |  |  |  |
|  | Limited in your ability to showering/bathing (reverse coded) | 3.95 | 1.2 | 4.33 | 1.07 | 0.33 |
|  | Limited in your ability to walking 1 block on level ground (reverse coded) | 2.58 | 1.46 | 3.18 | 1.49 | 0.41 |
|  | Limited in your ability to doing yardwork, housework, or carrying groceries (reverse coded) | 2.43 | 1.33 | 2.79 | 1.46 | 0.26 |
|  | Limited in your ability to climbing a flight of stairs without stopping (reverse coded) | 1.95 | 1.18 | 2.6 | 1.48 | 0.48 |
|  | Limited in your ability to hurrying or jogging (as if to catch a bus) (reverse coded) | 1.43 | 0.93 | 1.86 | 1.35 | 0.38 |
|  | Over the past 2 weeks, how many times did you have swelling in your feet, ankles, or legs when you woke up in the morning? (reverse coded) | 3.47 | 1.47 | 3.77 | 1.44 | 0.2 |
|  | Over the past 2 weeks, how much has your fatigue bothered you? (reverse coded) | 2.52 | 1.11 | 2.82 | 1.16 | 0.27 |
|  | If you had to spend the rest of your life with your heart failure the way it is right now, how would you feel about this? | 2.84 | 1.39 | 3.27 | 1.4 | 0.31 |
|  | Over the past 2 weeks, how often have you felt discouraged or down in the dumps because of your heart failure? (reverse coded) | 3.42 | 1.14 | 3.69 | 1.25 | 0.23 |
|  | Limitation to visiting family or friends outside of your home (reverse coded) | 3.38 | 1.36 | 3.81 | 1.31 | 0.32 |
